# Identification of cardiovascular risk factors among urban and rural Malaysian youths

**DOI:** 10.1186/s12872-021-02447-y

**Published:** 2022-02-23

**Authors:** Noor Shafina Mohd Nor, Yung-An Chua, Suraya Abdul Razak, Zaliha Ismail, Hapizah Nawawi, MyHEBAT-CRES investigators

**Affiliations:** 1 Institute of Pathology, Laboratory and Forensic Medicine (I-PPerForM), Selangor, Malaysia; 2 Faculty of Medicine, Universiti Teknologi MARA (UiTM), Sungai Buloh Campus, Selangor, Malaysia

**Keywords:** Coronary risk factors, Coronary artery disease, Youth, Urban, Rural

## Abstract

**Background:**

Coronary artery disease (CAD) is one of the major causes of morbidity and mortality worldwide. Early identification of the cardiovascular risk factors (CRF) among youths assists in determining the high-risk group to develop CAD in later life. In view of the modernised lifestyle, both urban and rural residing youths are thought to be equally exposed to various CRF. This study aimed to describe the common CRF including obesity, dyslipidaemia, hypertension, smoking and family history of hypercholesterolaemia and premature CAD in youths residing in urban and rural areas in Malaysia.

**Methods:**

We recruited 942 Malaysian subjects aged 15–24 years old [(males = 257, and urban = 555 vs. rural = 387, (mean age ± SD = 20.5 ± 2.1 years)] from the community health screening programmes organised in both rural and urban regions throughout Malaysia. Medical history and standardised anthropometric measurements were recorded. Laboratory investigations were obtained for fasting serum lipid profiles and plasma glucose levels.

**Results:**

A total of 43.7% from the total study population was either obese or overweight. Youths in the rural were more overweight and obese (49.4% vs. 42.7%, *p* < 0.044) and have higher family history of hypercholesterolaemia (16.3% vs. 11.3%, *p* < 0.036) than youths in the urban areas. Low-density lipoprotein (LDL-c) (2.8 vs. 2.7 mmol/L) and total cholesterol (TC) (4.7 vs. 4.5 mmol/L) were significantly higher in urban compared to rural youths (*p* < 0.019 and *p* < 0.012). Overall, more youth in this study has CRF rather than not (Has ≥ 1 CRF = 69.9%). Significantly more rural youths have at least one CRF compared to urban youths (rural = 74.2% vs. urban = 66.8%, *p* = 0.016).

**Conclusion:**

In conclusion, our study showed that a large number of youths had at least one or more CRF. Rural youths have significantly higher BMI with higher family history of hypercholesterolaemia compared to urban youths. However, urban youths have higher LDL-c and TC levels. Other coronary risk factors are not significantly different between urban and rural youths. Rural youths have more CRF compared to urban youths. A larger longitudinal study focusing on this population is important to better understand the effect of the area of residence on CRF in youth.

## Background

Coronary artery disease (CAD) is one of the major causes of morbidity and mortality worldwide. The World Health Organization (WHO) reported that an estimated 17.9 million people died from cardiovascular disease (CVD) in 2016 and of these deaths, 85% are due to CAD and stroke [1]. Majority of the deaths took place in low- and middle-income countries [1]. CAD had become a burden for the developing nations to date despite increase effort towards its prevention and treatment. In Malaysia, as one of the middle-income countries, CAD is the most common cause of deaths accounting for 98.9 deaths per 100,000 population in 2012, or 29,400 deaths (20.1% of all deaths) [2, 3].

Worryingly, there is a rapid emergence of increasing trend among younger Malaysians to present with CAD. Hoo et al. [4] reported that 6.1% of patients with Acute Coronary Syndrome (ACS) were less than 45 years of age with the mean age of young ACS in their study was 39 ± 6 years. The trend is consistent with other nearby South East Asian countries [5]. A study which retrospectively analysed 10,268 patients who underwent percutaneous coronary intervention in Malaysia reported a prevalence of young CAD of 16% (young was defined as less than 45 years for men and less than 55 years for women) [6].

There are multiple risk factors that are associated with progression of early CAD for instance hypertension, obesity, diabetes mellitus (DM), dyslipidaemia, physical inactivity and smoking [7–9]. In view of the recent trend of early CAD, prompt and early identification of the cardiovascular risk factors (CRF) among adolescents and youths is vital to assist in determining the high-risk group to develop CAD in later life. Urbanization in recent years may cause both urban and rural residing youths to be equally at risk of CVD. To date, there are very limited data reporting CRF in adolescents and youth population in this region. Furthermore, the difference of CRF between urban & rural Malaysian residents in this group is still not well documented. Hence, this study aimed to describe the common CRF including obesity, dyslipidaemia, hypertension, smoking and family history of hypercholesterolaemia and premature CAD in youths residing in urban and rural areas in Malaysia.

## Methods

### Study design and subject recruitment

This was a cross-sectional population-based study involving a total of 5448 Malaysian subjects from the community health screening programmes organised in both rural and urban regions in Malaysia. The participants were recruited via convenience sampling. The participation into the screening programmes was fully voluntary.

**Fig. 1:**
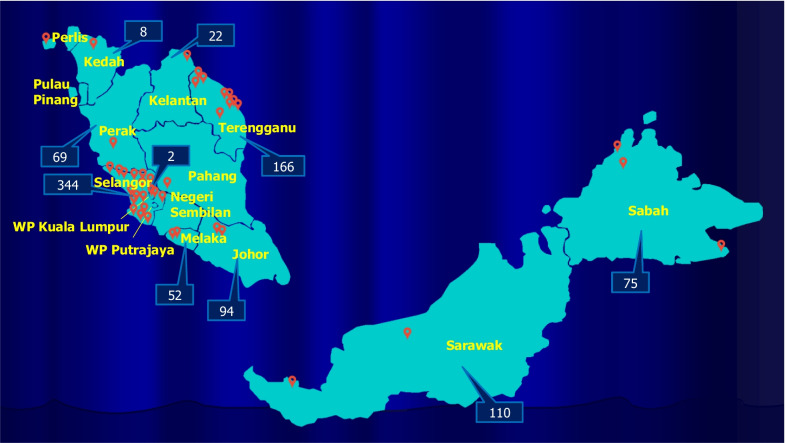
Map showing the screening sites and the number of participants included in this study

The health screening programme were held mainly in school halls or municipal general-purpose halls. The programmes were advertised by the local authorities via banners or social media approximately two weeks before the actual event. The recruitment was performed between 2011 to 2019. Following this, since this study focused on youth, a total of 942 youths aged between 15 and 24 years old (mean age ± SD = 20.5 ± 2.1 years) were included into the analyses. All of the data in this current study were from the original health screening programme. Figure 1 illustrated the map showing the screening sites and the number of participants included in this study. All regions of Malaysia namely, northern, eastern, western and southern part of Peninsular Malaysia as well as East Malaysia which included Sabah and Sarawak were included in the study in order to represent both urban and rural communities with the purpose of obtaining within-community homogeneity in the demographic profiles.

The inclusion criteria included Malaysian youths age between 15 and 24 years old [10]. Those with secondary hypercholesterolemia, acute inflammation, previous/current history of renal, liver, endocrine disease, morbid obesity (BMI > 35.0), patients on long term anti-inflammatory agents, chronic inflammatory disorders, life span-reducing disease including malignancy and those who were pregnant are excluded from the study.

**Fig. 2:**
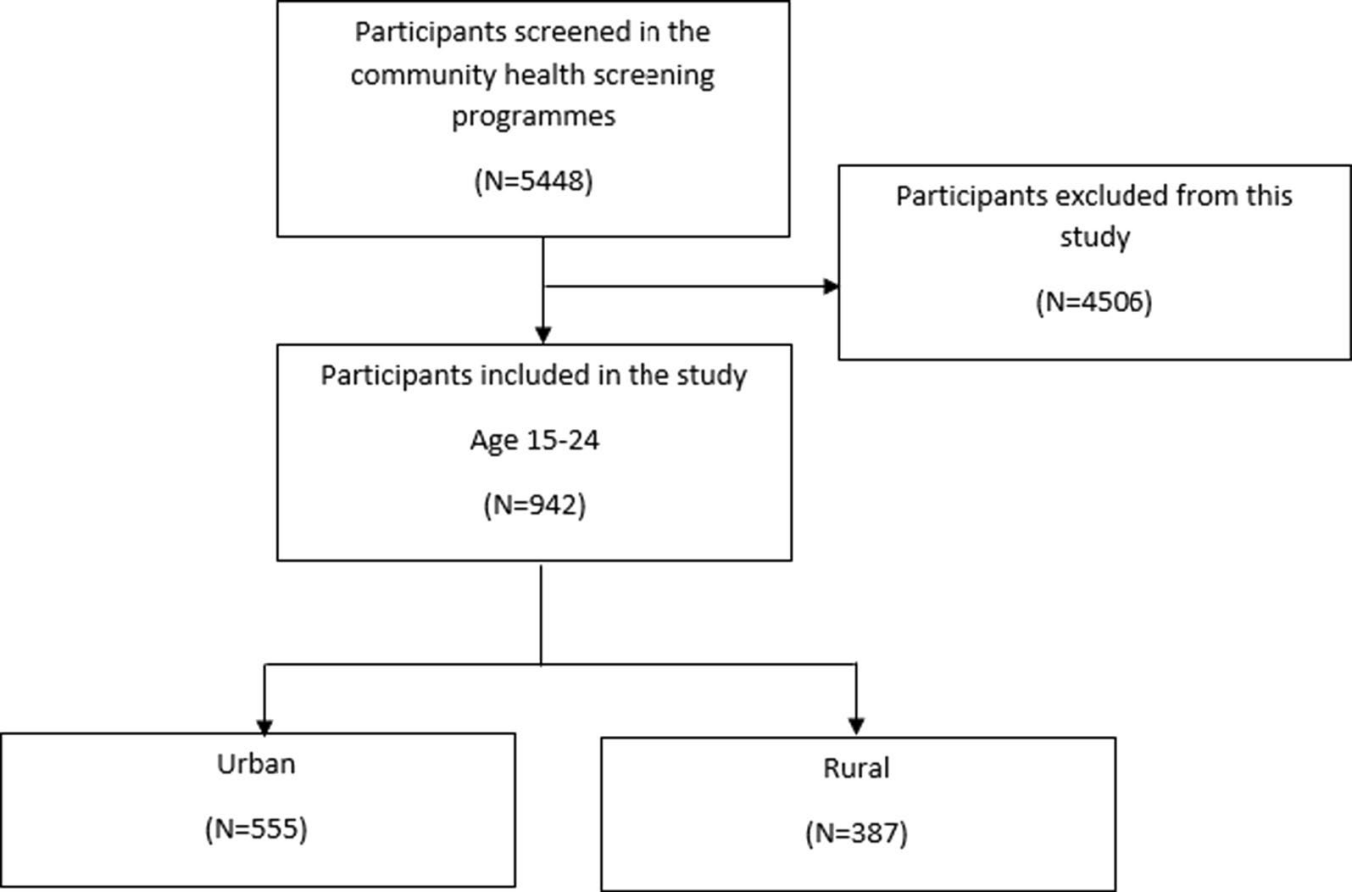
Flow chart depicting the cross-sectional study

Out of > 5000 participants screened, a total of 942 subjects met the enrolment criteria and therefore recruited. Subsequently, the recruited subjects were distinguished as urban (n = 555) and rural (n = 387) subject groups. Urban and rural areas were defined according to the Malaysian Population and Housing Census 2000 [11]. Urban areas were identified as areas with a combined population of 10,000 or more while all the other areas with a population of less than 10,000 were classified as rural. Figure 2 showed the flow chart of sample collection.

### Informed consent and ethical approval

The study was performed in accordance with the Declaration of Helsinki. All patients and parents gave their written informed consent. For those under 18 years old, informed consent were taken from their parents or legal guardian. The approval from the Institutional Research Ethics Committee [reference code: 600-RMI (5/1/6/01)] was obtained before the commencement of the study. All participants completed a standardised questionnaire which recorded the socio-demography and lifestyle which included age, gender, ethnic group, education level and smoking status, family history of premature CAD and hypercholesterolaemia.

### Anthropometry and definitions

All subjects were measured for their height, weight, body mass index (BMI), waist circumference, and blood pressure (BP) by trained professionals. Height and weight were measured to the nearest 0.1 cm and 0.1 kg respectively using a pre-calibrated freestanding mounted to scales stadiometer (Seca, Hamburg, Germany) and height rod in light clothing and without shoes on. WC was obtained using a measuring tape midway between the inferior margin of the rib and the topmost palpable border of the iliac crest with measurements taken to the nearest 0.5 cm.

BMI (kg/m^2^) was calculated as weight (kg) divided by squared height (m^2^). Obesity is defined as BMI ≥ 27.5 kg/m^2^ and overweight is defined as [12] BMI ≥ 23.0 kg/m^2^. On the other hand, Centers for Diseases Control and Prevention (CDC) BMI-to-age gender-specific percentile charts were used for subject aged 15–18 years old. For these population, overweight was defined as BMI equal or more than 85th percentile while obese was defined as BMI equal or more than 95th percentile [13].

An automated BP monitor (Omron, USA) was used to measure BP. The cuff was applied on the right arm held at heart level with the participants in seated position following at least 5-min rest. Three measurements were taken and the average of the last two readings were taken as the participant’s BP for the study. Elevated BP is defined as elevated BP with systolic BP (SBP) ≥ 130 mmHg and/or diastolic BP (DBP) ≥ 85 mmHg. On the other hand, for adolescents ages 15–17 years old, the systolic and diastolic hypertension were defined based on National Heart, Lung, and Blood Institute (NHLBI) guidelines for blood pressure in children and adolescent adapted from The Fourth Report on the Diagnosis, Evaluation and Treatment of High Blood Pressure in Children and Adolescent. Hypertension is defined as average SBP and/or diastolic BP (DBP) that is ≥ 95th percentile for gender, age, and height [14].

Data were collected by trained research assistants in order to minimise variability. The data were gathered via face-to-face interview using standardised validated questionnaire. These included the personal and family history of diabetes, hypertension, hypercholesterolemia and premature CAD with their medication intake. Smoking status and alcohol consumption were documented. Positive family history of premature CAD was defined as history of myocardial infarction, ischaemic heart disease or sudden death at the age of < 55 years in males and < 65 in females in first-degree relatives. Positive family history of hypercholesterolaemia were identified based on if their first-degree relatives have been diagnosed with hypercholesterolaemia (LDL-c > 4.9 mmol/L) or on lipid-lowering medications. The coronary risk factors included in this study were hypercholesterolaemia, low HDL, cigarette smoking, diabetes mellitus, hypertension, BMI overweight or above, central obesity, family history of hypercholesterolaemia and family history of premature CAD.

### Biochemical analyses

Fasting venous blood sample were collected into 5 mL of EDTA, serum and fluoride blood tubes after an overnight fast of at least 8 h. The serum and plasma were separated from blood samples within 2 h of collection by centrifugation at 1645 g for 7 min. The plasma were assayed for fasting plasma glucose (FPG) and serum were assayed for fasting serum lipid which consisted of total cholesterol (TC), triglycerides (TG), and high-density lipoprotein (HDL) using the hexokinase and enzymatic reference method respectively on an automated analyser (Cobas Integra 400 plus, Roche Systems, Germany). The Friedewald equation was used to determine low-density lipoprotein cholesterol (LDL-c) concentration. Hypercholesterolaemia was defined as LDL-c level of > 3.4 mmol/L. Low HDL was defined as HDL level < 1.0 and 1.3 mmol/L in males and females, respectively [15]. The intra- and inter-assay coefficient of variation (CV) for FPG, TC, TG and HDL were 1.8% and 2.1%; 0.5% and 1.9%; 1.6% and 1.9%; and 1.1% and 1.0%, respectively.

### Statistical analysis

All statistical analysis was performed using Statistical Program for Social Science Software (SPSS) version 23.0 (SPSS Inc. Chicago II, USA). Normally distributed continuous data were expressed as mean ± standard deviation (SD) or proportions (%), while non-normally distributed continuous data were expressed as median with interquartile range (IQR). Significant difference between groups with numerical data were analysed with t-test (normally distributed continuous data) or Mann–Whitney U test (non-normally distributed continuous data), while the association between categorical data was assessed using the chi-squared test. Risk model of CRF were calculated using binary logistic regression by analysing the number of CRF count against being urban or rural residents. The significant level in this study was set at *p* < 0.05.

## Result

The mean age for the total population was 20.5 ± 2.1 years. The baseline characteristics of the total population and according to group, either urban or rural are depicted in Table 1. The total study population were drug naïve where they were not on any anti-diabetic, anti-hypertensive, lipid-lowering agents and/or long-term antioxidant or anti-inflammatory therapy. A total of 43.7% from the total study population was either obese or overweight. Rural youths were significantly more overweight and obese (49.4% vs. 42.7%, *p* < 0.044) compared to urban youths. The mean BMI for total population, rural and urban were 23.2 ± 5.1, 23.5 ± 5.0 kg/m^2^ and 23.1 ± 5.3 kg/m^2^ respectively. Those who reside in rural area were also shown to have significantly higher family history of hypercholesterolaemia (16.3% vs. 11.3%, *p* < 0.036). Low-density lipoprotein (2.8 vs. 2.7 mmol/L) and total cholesterol (TC) (4.7 vs. 4.5 mmol/L) were significantly higher in urban compared to rural youths (*p* < 0.019 and *p* < 0.012 respectively) (Table 1). However, there were no differences or association between urban and rural youths in relation to HDL, TG, diabetes mellitus, smoking, hypertension, central obesity and family history of CAD. Urban and rural youths received almost equal opportunity of education, where the education levels were statistically not significant between each group (Table 2).

**Table 1:** Baseline characteristics for urban and rural youths (n = 942)

Variables	Urban (n = 555)	Rural (n = 387)	Total (n = 942)	*p* value*
Age (years) ± SD	20.2 ± 2.2	21.1 ± 1.7	20.5 ± 2.1	** < 0.001**
Male (n,%)	149 (26.8)	108 (27.9)	257 (27.3)	NS
Current smoker (n,%)	30 (5.4)	26 (6.7)	56 (5.9)	NS
BMI ± SD	23.1 ± 5.3	23.5 ± 5.0	23.2 ± 5.1	NS
Overweight (n, %)	140 (25.2)	116 (30.0)	256 (27.2)	NS
Obese (n, %)	87 (15.7)	69 (17.8)	156 (16.6)	NS
Overweight and obese (n,%)	227 (40.9)	185 (47.8)	412 (43.7)	** < 0.038**
Central obesity (n,%)	150 (27.0)	96 (24.8)	246 (26.1)	NS
Diabetes mellitus (n,%)	1 (0.2)	1 (0.8)	2 (0.2)	NS
Hypertension (n,%)	3 (0.5)	2 (0.5)	5 (0.5)	NS
Family history hypercholesterolaemia (n,%)	64 (11.5)	63 (16.3)	127 (13.5)	** < 0.036**
Family history PCAD (n,%)	45 (8.1)	45 (11.6)	90 (9.6)	NS
LDL-c (mmol/L) ± SD	2.8 ± 0.8	2.7 ± 0.8	2.8 ± 0.8	** < 0.019**
TC (mmol/L) ± SD	4.7 ± 0.9	4.5 ± 1.0	4.6 ± 0.9	** < 0.012**
TG (mmol/L) (IQR)^a^	0.9, (0.6)	1.0 (0.6)	1.0, (0.6)	NS
HDL (mmol/L) ± SD	1.4 ± 0.3	1.4 ± 0.3	1.4 ± 0.3	NS
Glucose (mmol/L) ± SD	5.0 ± 1.1	5.1 ± 1.5	5.1 ± 1.3	NS

*Categorical data were analysed with chi-squared. Continuous data were analysed with independent t-test, unless stated otherwise (Significantly different: *p* < 0.05)

LDL-c, low-density lipoprotein cholesterol; TC, total cholesterol; TG, triglycerides; HDL, high-density lipoprotein; NS, not significant; IQR, interquartile range

^a^Mann–Whitney U test

**Table 2:** Education levels among Malaysian urban and rural youths (n = 827)

Education level	Urban (N = 480)^a^ N (%)	Rural (N = 347)^a^ n (%)	Total (N = 827)^a^ n (%)	*p* value*
Primary school or lower	3 (0.6)	2 (0.6)	5 (0.6)	NS
Secondary school (PT3- SPM)	106 (22.1)	63 (18.2)	169 (20.4)	NS
Diploma (STPM)	217 (45.2)	177 (51.0)	394 (47.6)	NS
Bachelor degree	152 (31.7)	99 (28.5)	251 (30.4)	NS
Post-graduate	2 (0.4)	6 (1.7)	8 (1.0)	NS

NS, not significant

*Chi-squared analysis, significant association (*p* < 0.05) between urban and rural youths

^a^Subjects without data of education level were excluded

Overall, more youth in this study has CRF rather than not (658/942, 69.9% have CRF). Significantly more rural youths have at least one CRF compared to urban youths (rural = 74.2% vs. urban = 66.8%, *p* = 0.016) (Table 3). Generally, rural youth individuals tend to have more CRF compared to urban youth (mean number of CRF 1.52 vs. 1.36), where the maximum number of CRF in rural youths was six, compared 5 CRF in urban youths. Due to the similarity of education opportunity between rural and urban youths, the education level might have equal effects on the CRF in both youth groups. Subjects with lower education level have 11 times higher chance of having low HDL [OR 11.364 (CI 1.263–102.241, *p* = 0.006)], while subjects with higher education level have lesser chance of having low HDL [OR 0.670 (CI 0.473–0.950, *p* = 0.024)]. Family history of hypercholesterolaemia and premature CAD were also associated with secondary [OR 0.523 (CI 0.351–0.780, *p* = 0.001) and OR 0.623 (CI 0.388–1.000, *p* = 0.048), respectively] and tertiary education [OR 1.820 (CI 1.218–2.718, *p* = 0.003) and OR 1.657 (CI 1.032–2.661, *p* = 0.035), respectively], with a trend of higher odds ratio among those with higher education.

**Table 3:** The number of coronary risk factors among rural and urban youths (n = 942)

Number of coronary risk factors (CRF)^a^	Total (N = 942) n (%)	Urban (N = 555) n (%)	Rural (N = 387) n (%)	Chi-squared^a^	*p* value*	Odds ratio (95% CI)^b^
1 or more	658 (69.9%)	371 (66.8%)	287 (74.2%)	χ^2^ (1) = 5.791	**0.016**	**1.423 (1.067–1.899)**
1	265 (28.1%)	146 (26.3%)	119 (30.7%)	χ^2^ (1) = 5.373	**0.020**	**1.500 (1.064–2.114)**
2	185 (19.6%)	109 (19.6%)	76 (19.6%)	χ^2^ (1) = 1.646	NS	1.283 (0.877**–**1.878)
3	140 (14.9%)	79 (14.2%)	61 (15.8%)	χ^2^ (1) = 2.783	NS	1.421 (0.940**–**2.148)
4	54 (5.7%)	32 (5.8%)	22 (5.7%)	χ^2^ (1) = 0.601	NS	1.265 (0.698**–**2.293)
5	12 (1.3%)	5 (0.9%)	7 (1.8%)	χ^2^ (1) = 2.667	NS	2.576 (0.797**–**8.326)
6	2 (0.2%)	0 (0%)	2 (0.5%)	NA	NA	NA
Mean number of CRF ± SD	1.43 ± 1.3	1.36 ± 1.3	1.52 ± 1.2	–	0.057^c^	–

NS, not significant; NA, not applicable due to insufficient individual count for chi-squared analysis

*Chi-squared analysis between urban or rural youths with CRF (significant association *p* < 0.05). Chi-squared analysis was performed between the number of CRF (eg: one CRF or no CRF, two CRF or no CRF) against urban or rural

^a^For chi-squared analysis, the numbers of CRF (Yes) were constantly paired with zero CRF (No). The rows for zero CRF were not displayed

^b^Binary logistic regression analysis. Dependent variables: number of CRF. Independent variable: rural/urban youths

^c^t-test

Coronary risk factors: Hypercholesterolaemia (LDL-c > 3.4 mmol/L), low HDL (males < 1.0 mmol/L, female < 1.3 mmol/L), current smoker, diabetes mellitus (fasting glucose < 7.0 mmol/L), hypertension (systolic blood pressure > 130 mmHg and/or diastolic blood pressure > 85 mmHg), body mass index = overweight or obese, central obesity (waist circumference: males > 90 cm, females > 80 cm), family history of hypercholesterolaemia (LDL-c > 4.9 mmol/L), family history of premature coronary artery diseases.

## Discussion

Our current study evaluated the CRF in youth population and compared them between the urban and rural youths. To the best of our knowledge, the data that reported on this group of young adults is very scarce especially when comparing between the area of residence. Our study showed that a large number of youths in the total population had at least one or more CRFs. CRF were significantly more prevalent among rural compared to urban youths, and the outcomes concurred with the Malaysian previous general population study [16, 17]. They reported that smoking, obesity, hypertension, diabetes and depression were identified as more prevalent among rural residents compared to urban [16]. Increasing urbanisation and modernisation may be one of the major causes to influence the more prevalent CRF in rural. The lifestyles changes in this population may have contributed to the current trend in our country and some other countries.

Prevalence of CRF between urban and rural populations are largely conflicting worldwide, and many factors may contribute to this for instance the countries’ Gross Domestic Product (GDP). Based on the Malaysian Report on Household Income and Basic Amenities Survey 2019, the mean income for urban was RM8635, while in rural area the mean household income was RM5004. Smoking, obesity, hypertension, diabetes and hyperlipidaemia, were more rampant in rural population of certain countries like Sweden and India [18, 19], but reported to be on the contrary in other countries like China, Ghana and Peru [20–22]. Ezzati et al. reported that CRF are expected to shift to low-income and middle-income countries and, with the persistent heavy burden of infectious diseases in these countries will further increase the global health inequalities [23]. Similarly, Rabin et al. stated that GDP and obesity have a negative association in high-income European countries [24]. However, another study did not find any relationship between CRF (obesity, insufficient activity, systolic blood pressure, and fasting plasma glucose) with GDP [25]. Comparably, Danaei et al. indicated that a country’s GDP level does not indicate that there must be health behavior change and health improvement endeavors and suggested that the countries’ income has a rather indirect relationship with health behavior or health improvement endeavors [26]. However, majority of the aforementioned studies were conducted among general population without age grouping. The differences in socioeconomic, lifestyle and stress exposure may affect the prevalence of CRF between general population and youths.

Our study showed that rural group was significantly more overweight and obese, and has higher prevalence of family history with hypercholesterolaemia. This trend is interesting and also alarming at the same time showing that obesity is not entirely associated with the place of residence and urbanisation or modernisation may play an important role [27]. In one study among 16–35 years old participants in a district in Malaysia, Pell et al. reported that the prevalence of overweight was 12.8% at ages 16–20 and 28.4% at ages 31–35 while obesity was 7.9% and 20.9% at the same age group respectively [28]. However, since it was only from one district, a comparison between rural and urban was not performed. They also highlighted that the pattern among this age group suggests that this is a significant period for change in health-related behaviours [28]. Other Malaysian studies looking at younger age group among the children and adolescents reported that the rural group had higher odds of overweight and obesity suggesting that rural environment may be more “obesogenic” in ways that a person-level analysis is unable to distinguish [29]. On contrary to another recent publication which reported that there was no significant difference in BMI status between rural and urban as well as between genders [30]. Interestingly, our neighboring country Indonesia reported that the prevalence of obesity was higher in urban children and adolescents compared to rural [31]. In rural Indonesia however, participants with higher salty and grilled foods intake had higher risk for obesity and overweight [31]. However, direct comparison is difficult due to the difference in the study group between their population and ours.

Despite the majority of CRF were more prevalent in rural youths in our study, urban youths had significantly higher LDL-c and TC compared to rural youths. Worse lipid profile is probably associated with high-fat diet or fast food which are more widely available in the city. Contrary to our findings, Nuotio et al. [32] reported that Finnish living in urban areas had lower levels of LDL-c and Hba1c, and lower rate of metabolic syndrome (MS) compared to rural residents, albeit without restricting the age of the subjects. These contradicting findings leave us the question of what is the factor that dictate the CRF among youths, if the location of residency is not the determining factor. A study in Indian young adult population (age 26–32 years) had attempted to associate Household Possession Score, individual education and paternal education status with CRF. The study consistently found that subjects with higher score of these socioeconomic status indicators were associated with more CRF [33], regardless whether they are rural or urban residents, hypothesising that CRF is related to greater accessibility to food and less physically-straining job among higher socioeconomic status people.

A shift in the association of risk factor level and socioeconomic status could also explain the different findings found between low-income and high-income countries. Therefore, higher CRF rate in rural resident in this present study may be an indicator that rural lifestyle does not necessarily representing lower socioeconomic status. Malaysian residents between the age range of this study cohort are probably fresh graduates who are migrating from their hometown to big cities seeking for jobs, where their first jobs are probably more physically taxing with less leisure time. On the contrary, rural youth residents in this study are probably in a higher socioeconomic status background who do not need to eke out their living and enjoyed more sedentary lifestyle at countryside. Furthermore, at the same time they might also be exposed to obesogenic food sources due to the gradual modernisation of Malaysian towns.

If the main contributor of CRF among youths is socioeconomic status, the future preventive measure in preventing premature CVD death should focus in spreading awareness of healthy lifestyle among these high socioeconomic status youths, regardless if they are urban or rural residents. Higher education level can be an indicator of higher socioeconomic status, but since education coverage of Malaysian youths in this study are quite uniform, additional information, such as household income, are required to deduce the cause of CRF among this group. Rosengren et al. [34] showed that the differences in the outcomes among different education levels were not defined by the differences in the risk factors. The risk decreased as the level of education increased in the high-income countries while in low-income countries it increased. Our study showed that subjects with lower education have 11 times higher chance of having low HDL. Those with higher education level in our study also were shown to have higher family history of hypercholesterolaemia and premature CAD. The other risk factors were not significant between the different education levels. However, some other studies in the Malaysian general population showed that better education is associated with less CRF, such as obesity [35]. Our study found that other CRF like HDL, TG, diabetes mellitus, smoking, hypertension, central obesity and family history of CAD are not significantly different between urban and rural youths.

In addition, our study showed a larger number of females compared to males. The possible explanation for this was females who were not working or were housewives were more likely able to attend the health screening programmes compared to males who are usually working. This could be one of the possible explanations. However, the gender proportions between urban and rural groups were not statistically significant. Similarly, Ismail et al. [16] also reported more females in their total study population. They also reported similar findings whereby rural areas generally have higher CVD risk factors due to ageing, lower educational status and unhealthy diet. However, this study included older population between 35 and 70 years of age unlike our younger sample population. The fact that this study had more females compared to males also may contribute to smaller proportion of smokers in our study compared to the other study [18].

Our study showed no significant difference between urban and rural in relation to hypertension. However, this contradict the finding from another local study whereby hypertension was shown to be significantly more prevalent in the rural population [17]. Rural population was also reported to have hypertension less controlled with anti-hypertensive medication. However, this study included participants from 30 years of age with a mean age of 53 years old, which is a stark contrast compared to our much younger subjects whose cardiovascular events such as age-dependent blood vessel stiffening was still not fully developed.

A significant strength of our study is the recruitment of a large number of participants from youth population. They were still not fully exposed to unhealthy lifestyle or therapeutic intervention, ie drug naïve, where they were not on anti-diabetic, anti-hypertensive, lipid-lowering agents and/or long-term antioxidant or anti-inflammatory therapy which may act as confounders. This study also compared between the urban and rural youth population which data is still scarce to date. This information is highly valuable especially within this Asian region in assisting to better understand this group of patients. However, a perceived limitation is that although the study compared between the urban and rural youth population in all regions of Malaysia including northern, eastern, western and southern part of Peninsular Malaysia as well as East Malaysia which included Sabah and Sarawak, this may still not reflect the true representation for this population. The definition of urban and rural was somehow subjective and based on the classification gazetted by the local authorities. Furthermore, since this study is cross-sectional and hence is only able to demonstrate association rather than causal effect. The socioeconomic status of the subjects was also not being fully factored into the association with CRF in this study. Some other information regarding physical activity, psychological issue, HbA1c and c-reactive protein levels were also not available in this study. Our study also clearly showed a higher female participation with low rate of smoking which may indicate a selection bias in the sample selection. Studies have shown that factors which are linked to nonparticipation in health checks include male gender, young age group, smoking and other cardiovascular risk factors as well as socio-demographic status [36]. This fact may indicate that the situation in the real population is likely to be worse compared to what was seen in this present study due to the selection bias. Hence, this mandates serious action to be taken by all the relevant stakeholders to reduce future health crisis. Moreover, problems may arise in comparison between these two groups, when there is uncertainty as to whether the selection is similar in the rural and urban areas. Therefore, future longitudinal studies concentrating on this group of youths are vital to better understand the nature of the coronary risk profiles and aid in reducing the prevalence of CAD in later life.

## Conclusion

In conclusion, our study showed that a large number of youth population had at least one or more CRF. Rural youths are significantly heavier with higher family history of hypercholesterolaemia compared to urban youths, while urban youths have higher LDL-c and TC levels. Other cardiovascular risk factors are not significantly different between urban and rural youths. Generally, rural youths have more CRF than urban youths, but the cause of this in this youth group is still unclear. Healthy lifestyle awareness and intervention programmes are pivotal in these targeted groups of youths to reduce the CHD risk in later life. However, in view of the study limitations, a larger longitudinal study focusing on this population is important to better understand the effect of the area of residence on CRF in youth, to better inform strategic policies and actions, in the prevention of future premature CHD in these youths. The fact that the situation in the real population is likely to be worse compared to what has been reported in this present study, warrants a stronger need for action from the stakeholders.

## APPENDIX 1

MyHEBAT - CRES (**M**ala**y**sian **HE**alth and Well-**B**eing **A**ssessmen**T** - **C**oronary **R**isk **E**pidemiological Study) investigators:

^1^**Institute of Pathology, Laboratory and Forensic Medicine (I-PPerForM) and** ^2^**Faculty of Medicine, Universiti Teknologi MARA (UiTM):** Hapizah Nawawi, Anis Safura Ramli, Suraya Abdul Razak, Noor Alicezah Mohd Kasim, Thuhairah Hasrah Abdul Rahman, Effat Omar, Noor Shafina Mohd Nor, Alyaa Al-Khateeb, Suhaila Abd Muid, Mansharan Kaur Chainchel Singh, Yung-An Chua, Nurul’Ain Abu Bakar, Aimi Zafira Razman, Sukma Azureen Nazli, Amirah Mohd Arif, Fadlullah Jili Fursani Kemrry.

**Institute of Pathology, Laboratory and Forensic Medicine (I-PPerForM) and Faculty of Dentistry, Universiti Teknologi MARA (UiTM):** Mohd Yusmiaidil Putera Mohd Yusof.

**Faculty of Medicine, Universiti Teknologi MARA (UiTM):** Zaliha Ismail, Norashikin Mohd Ranai, Ahmad Taufik Jamil, Ahmad Bakhtiar Md Radzi, Khairul Shafiq Ibrahim.

**Faculty of Medicine, Universiti Malaya:** Mohd Amin Jalaludin.

**Faculty of Medicine and Health Sciences, Universiti Sultan Zainal Abidin (UniSZA):** Ahmad Zubaidi Abd Latif.

**Faculty Of Medicine, Universiti Kuala Lumpur (UniKL) Royal College of Medicine Perak (RCMP):** Osman Ali.

## Data Availability

The datasets used and/or analysed during the current study are available from the corresponding author (hapizah.nawawi@gmail.com).

## Abbreviations

ACS: Acute coronary syndrome
BMI: Body mass index
BP: Blood pressure
CVD: Cardiovascular disease
CAD: Coronary artery disease
CRF: Coronary risk factor
LDL-c: Low-density lipoprotein cholesterol
TC: Total cholesterol
TG: Triglycerides
HDL: High-density lipoprotein

## References

[1] Cardiovascular diseases (CVDs) [Internet]. Geneva (CH): World Health Organization: 2021. Available from: https://www.who.int/en/news-room/fact-sheets/detail/cardiovascular-diseases-(cvds).

[2] World Health Organization. Country statistics and global health estimates, 2015.

[3] Institute for Public Health Ministry of Health Malaysia. Malaysian burden of disease and injury study: 2009–2014. Kuala Lumpur: 2017.

[4] Hoo FK, Foo YL, Lim SMS, Ching SM, Boo YL (2016). Acute coronary syndrome in young adults from a Malaysian tertiary care centre. Pak J Med Sci..

[5] Tungsubutra W, Tresukosol D, Buddhari W, Boonsom W, Sanguanwang S, Srichaiveth B (2007). Thai ACS Registry: acute coronary syndrome in young adults: the Thai ACS Registry. J Med Assoc Thai.

[6] Zuhdi AS, Mariapun J, Mohd Hairi NN, Wan Ahmad WA, Abidin IZ, Undok AW (2013). Young coronary artery disease in patients undergoing percutaneous coronary intervention. Ann Saudi Med.

[7] Khot UN, Khot MB, Bajzer CT (2003). Prevalence of conventional risk factors in patients with coronary heart disease. JAMA.

[8] Joseph P, Leong D, Mckee M (2017). Reducing the global burden of cardiovascular disease, part 1: the epidemiology and risk factors. Circ Res.

[9] Poorzand H, Tsarouhas K, Hozhabrossadati SA, Khorrampazhouh N, Bondarsahebi Y, Bacopoulou F (2019). Risk factors of premature coronary artery disease in Iran: a systematic review and meta-analysis. Eur J Clin Invest.

[10] United Nations. https://www.un.org/en/sections/issues-depth/youth-0/index.html

[11] Malaysia Department of Statistic. Population distribution and basic demographic characteristics. 2000, Dept of Statistics, Malaysia Dept of Statistics Malaysia.

[12] Zainudin S, Daud Z, Mohamad M, Tan TB, Wan Mohamed WMI (2011). A summary of the Malaysian clinical practice guidelines on management of obesity 2004. J ASEAN Fed Endocr Soc.

[13] Rosner B, Prineas R, Loggie J, Daniels SR (1998). Percentiles for body mass index in US children 5 to 17 years of age. J Pediatr.

[14] Blood Pressure levels for boys and girls by age and height percentile.

[15] Raanan S, Lerner A, Fisher E (2000). Hypercholesterolemia in children. Isr Med Assoc J (IMAJ).

[16] Ismail NH, Rosli NM, Mahat D, Yusof KH, Ismail R (2016). Cardiovascular risk assessment between urban and rural population in Malaysia. Med J Malays.

[17] Abdul-Razak S, Mohammad Daher A, Ramli AS, Ariffin F, Md Yasin M, Ambigga S (2016). Prevalence, awareness, treatment, control and socio demographic determinants of hypertension in Malaysian adults. BMC Public Health.

[18] Sweta B, Devi Radha B (2018). Risk factors of cardiovascular disease between urban and rural adult population. Int J Caring Sci.

[19] Lindroth M, Lundqvist R, Lilja M, Eliasson M (2014). Cardiovascular risk factors differ between rural and urban Sweden: the 2009 Northern Sweden MONICA cohort. BMC Public Health.

[20] Kodaman N, Aldrich MC, Sobota R, Asselbergs FW, Poku KA, Brown NJ (2016). Cardiovascular disease risk factors in Ghana during the rural-to-urban transition: a cross-sectional study. PLoS ONE.

[21] Yan R, Li W, Yin L, Wang Y, Bo J, Investigators PC, Investigators PC, Liu L, Liu B, Hu B (2017). Cardiovascular diseases and risk-factor burden in urban and rural communities in high-, middle-, and low-income regions of china: a large community-based epidemiological study. J Am Heart Assoc.

[22] Miranda JJ, Gilman RH, Smeeth L (2011). Differences in cardiovascular risk factors in rural, urban and rural-to-urban migrants in Peru. Heart.

[23] Ezzati M, Vander Hoorn S, Lawes CMM, Leach R, James WPT, Lopez AD (2005). Rethinking the “diseases of affluence” paradigm: global patterns of nutritional risks in relation to economic development. PLoS Med.

[24] Rabin BA, Boehmer TK, Brownson RC (2006). Cross-national comparison of environmental and policy correlates of obesity in Europe. EUR J Public Health.

[25] Dadgar I, Norström T (2020). Is there a link between cardiovascular mortality and economic fluctuations?. Scand J Public Health.

[26] Danaei G, Singh GM, Paciorek CJ, Lin JK, Cowan MJ, Finucane MM (2013). The global cardiovascular risk transition associations of four metabolic risk factors with national income, urbanization, and western diet in 1980 and 2008. Circulation.

[27] Ismail MN, Chee SS, Nawawi H, Yusoff K, Lim TO, James WPT (2002). Obesity in Malaysia. Obes Rev.

[28] Pell C, Allotey P, Evans N (2016). Coming of age, becoming obese: a cross-sectional analysis of obesity among adolescents and young adults in Malaysia. BMC Public Health.

[29] Liu JH, Jones SJ, Sun H, Probst JC, Merchant AT, Cavicchia P (2012). Diet, physical activity, and sedentary behaviors as risk factors for childhood obesity: an urban and rural comparison. Child Obes.

[30] Ahmad A, Zulaily N, Shahril MR, Syed Abdullah EFH, Ahmed A (2018). Association between socioeconomic status and obesity among 12-year-old Malaysian adolescents. PLoS ONE.

[31] Nurwanti E, Hadi H, Chang JS, Chao JCJ, Paramashanti BA, Gittelsohn J, Bai CH (2019). Rural-urban differences in dietary behavior and obesity: results of the riskesdas study in 10–18-year-old indonesian children and adolescents. Nutrients.

[32] Nuotio J, Vähämurto L, Pahkala K, Magnussen CG, Hutri-Kähönen N, Kähönen M (2019). CVD risk factors and surrogate markers—urban-rural differences. Scand J Public Health.

[33] Samuel P, Antonisamy B, Raghupathy P, Richard J, Fall CH (2012). Socio-economic status and cardiovascular risk factors in rural and urban areas of Vellore, Tamil Nadu, South India. Int J Epidemiol.

[34] Rosengren A, Smyth A, Rangarajan S, Ramasundarahettige C, Bangdiwala SI, AlHabib KF, Avezum A, Bengtsson Boström K, Chifamba J, Gulec S, Gupta R, Igumbor EU, Iqbal R, Ismail N, Joseph P, Kaur M, Khatib R, Kruger IM, Lamelas P, Lanas F, Lear SA, Li W, Wang C, Quiang D, Wang Y, Lopez-Jaramillo P, Mohammadifard N, Mohan V, Mony PK, Poirier P, Srilatha S, Szuba A, Teo K, Wielgosz A, Yeates KE, Yusoff K, Yusuf R, Yusufali AH, Attaei MW, McKee M, Yusuf S (2019). Socioeconomic status and risk of cardiovascular disease in 20 low-income, middle-income, and high-income countries: the Prospective Urban Rural Epidemiologic (PURE) study. Lancet Glob Health.

[35] Lim TO, Ding LM, Zaki M, Suleiman AB, Fatimah S, Siti S (2000). Distribution of body weight, height and body mass index in a national sample of Malaysian adults. Med J Malays.

[36] Dryden R, Williams B, McCowan C (2012). What do we know about who does and does not attend general health checks? Findings from a narrative scoping review. BMC Public Health.

